# Two Cases of Persistent Complex Bereavement Disorder Diagnosed in the Acute Inpatient Unit

**DOI:** 10.1155/2020/3632060

**Published:** 2020-04-02

**Authors:** Olalekan Olaolu, Terrence Tumenta, Samuel Adeyemo, Olusegun Popoola, Oluwatoyin Oladeji, Tolu Olupona

**Affiliations:** Department of Psychiatry and Behavioral Health, Interfaith Medical Center, 1545 Atlantic Avenue, Brooklyn, New York 11213, USA

## Abstract

Pathological grief has been noted to have considerable adverse effects on affected individuals. In the DSM-5, the diagnosis of complicated grief is included under conditions for further study as Persistent Complex Bereavement Disorder (PCBD). PCBD can be easily missed because it is a relatively new and developing diagnosis. It can also be overlooked when it is comorbid with more common psychiatric disorders. We present 2 patients with PCBD diagnosed in the inpatient unit, while the patients were admitted for comorbid disorders. PCBD contributed immensely to both patients' suffering and decline in functioning. This report highlights the presentation, diagnoses, and management of these patients. We theorize that paying attention to separation distress, reactive distress to loss, and identity disruption in individuals who have been bereaved for over 12 months will enhance treatment specificity and lead to better patient outcomes.

## 1. Introduction

Persistent Complex Bereavement Disorder (PCBD) is included as a condition for further study in the 5^th^ edition of the Diagnostic and Statistical Manual of Mental Disorders [1]. The proposed PCBD criteria comprise 16 symptoms organized in three clusters, namely, separation distress, reactive distress to the death, and social/identity disruption [1]. A diagnosis of PCBD requires that the person has experienced the death of someone with whom he/she had a close relationship, and the endorsement of at least one separation distress symptom and six additional symptoms. Additionally, these symptoms must be associated with functional impairment and have persisted for at least 12 months (6 months for children) after the death [1]. Similarly, the World Health Organization has proposed adding Prolonged Grief Disorder (PGD) to the upcoming 11^th^ edition of the International Classification of Diseases (ICD-11) [2]. For PGD, one out of two separation distress symptoms combined with at least one out of ten accompanying symptoms present 6 months after bereavement is required for diagnosis [2].

There are three other criteria used in literature to define pathological grief. They include Prolonged Grief Disorder by Prigerson et al., Complicated Grief by Shear et al., and the beta draft of the ICD-11 criteria [3]. Prolonged Grief Disorder by Prigerson et al. [4] requires separation distress and the presence of five of nine additional cognitive/emotional/behavioral symptoms 6 months postbereavement. Complicated Grief by Shear et al. [5] requires one symptom of separation distress and 2 additional symptoms of 1-month duration occurring 6 months postbereavement. The beta draft of the ICD-11 criteria is an early version of the ICD-11 PGD criteria. All criteria require clinically significant distress. There is significant overlap between all five criteria [3]. In these 2 cases, we used the PCBD criteria from the DSM-5.

Pathological grief is often seen in about 7–20% of bereaved individuals [6, 7]. It is more commonly encountered in the psychiatric outpatient setting [7]. Simon [6] in a report on treating complicated grief comprehensively classified risk factors for PCBD into preloss, loss-related, and periloss factors (see Table 1). PCBD is associated with impaired quality of life [6, 7]; other psychiatric comorbidities including major depressive disorder (MDD) [7], posttraumatic stress disorder (PTSD) [8], generalized anxiety disorder (GAD) [8], and panic disorder [8]; and chronic physical illnesses [6, 7]. It is important to note that while PCBD may be comorbid with other psychiatric illnesses, it can also be a primary disorder which can cause significant physical and mental dysfunction [9–12], hence the need to promptly identify this condition and treat appropriately.

The underlying biological disorder in PCBD remains unknown, but several neural mechanisms have been implicated. Likewise, the treatment of PCBD is emerging. Both the neurobiology and treatment of PCBD are further reviewed in Discussion.

We present 2 cases, a 41-year-old Hispanic female and a 19-year-old African American male who were diagnosed with PCBD while admitted to the acute psychiatric inpatient unit of a community teaching hospital.

## 2. Case Presentation

### 2.1. Case 1

AB, a 41-year-old Hispanic female, single, employed, and domiciled alone in an apartment, was brought into the emergency department (ED) by EMS on account of bizarre behavior. Per reports, the patient was at a restaurant with friends when she suddenly started screaming, being uncharacteristically talkative, and stating that she wanted to hurt herself.

On evaluation in the ED, AB was uncooperative and irritable. She was crying uncontrollably throughout her interview. She stated that she had not been feeling well in the past week and was in a bar drinking with friends. She could not remember the circumstances that led to her presentation in the hospital. She described her mood as “sad and angry.” Her affect was labile. Alcohol level was 195 mg/dl. Other labs and EKG were within normal limits. Patient was admitted for safety and stabilization.

During the inpatient evaluation, AB reported being extremely stressed over her son's death from cancer at age 14, four years before current presentation. She described her son as everything to her and believed life was meaningless without him. She described feeling traumatized after his death, and she became hypervigilant to cancer or cancer-related topics. She also reported having consistent nightmares of her son drowning and being unable to save him.

Since his death, she has had to change jobs and move back to the city where she grew up to be in touch with her family for support. At her current job, she was given extended time away “to take care of herself.” She avoided business opportunities if they emanated from people working in fields related to cancer. She also reported becoming excessively reactive when witnessing adverse human experiences—like famine, natural disasters, and accidents—either in person or through the media. Her reactions to suffering consisted of episodes of jitteriness, excessive crying, and tingling fingers.

Two months prior to her admission, she voluntarily presented to the ED at another hospital with somatic symptoms (jitteriness and tingling fingers) and lower neck pain, radiating down her left arm. At the time, she feared she was having a heart attack. After evaluation, she was informed that she was stressed and did not have any cardiac abnormalities. She subsequently presented to a primary care physician where she was diagnosed with PTSD.

She reported the daily use of marijuana (3 blunts per day) and tobacco (7 cigarettes per day) beginning at ages 25 and 27, respectively. She reported being a social drinker. Per collateral information from the patient's friend of 25 years, patient had led a relatively normal life until her son's death, and due to her unrelenting reaction to the death, the entire family has been worried about the patient. Collateral information revealed that though patient was a social drinker, she had begun drinking more in months before presentation as a coping mechanism and consequently had had three blackouts in the 6-month period before presentation.

Patient reported past psychiatric hospitalization for 30 days at age 16 for suicide attempt by an overdose. She was diagnosed with MDD at the time. She stopped using medications after discharge and did not follow up with aftercare at the time.

The patient was discharged on the 2^nd^ day to family members on mirtazapine and referred for therapy.

### 2.2. Case 2

XY, a 19-year-old single African American male, undomiciled, and unemployed (supported by extended family), enrolled in a vocational training program and walked into the ED requesting medication refill.

On evaluation, the patient was found to be severely depressed, apathetic, and nihilistic. His labs and EKG were within normal limits. He was admitted for safety and stabilization.

During inpatient evaluation, the patient reported that his mother died of cancer 4 years prior to presentation when he was 15 years old. He reported being by his mother in the hospital when she died. He blamed himself for his mother's death and reported feeling guilty, lost, and sad about his mother's passing: “I wish I could have been a better son. I wish I could have done more for her.”

Since his mother's death, he has been yearning for her, longing to be with her, and has had frequent crying spells. The crying spells were often prompted by flashbacks of his mother, about six times a week. He reported repeated nightmares in which his mother calls him, but he cannot answer. He states that he usually wakes up from such dreams drenched in sweat. The patient also started smoking marijuana which prompted the rest of his family to abandon him, further hollowing his support structure.

He reported difficulty with sleep (three hours of sleep at night) and loss of interest in playing his saxophone and trumpet and playing sports. He reported low energy and poor appetite for about five months. He denied suicidal or homicidal ideation. The patient was overwhelmed by his symptoms and had to be placed on medical leave from his professional training program. Collateral information from his training program revealed that the patient's “mental health was interfering with his work.” Beck's Depression Inventory score was 19 (a score between 17 and 20 indicates borderline clinical depression) [14], and the Hamilton Depression Rating Scale (HAM-D) score was 21 (a score between 19 and 22 indicates severe depression) [14].

Six months prior to presentation, he was diagnosed with MDD and hospitalized for one month for suicidal ideation. He was managed with bupropion and buspirone. Three months prior to presentation, the patient was diagnosed with PTSD and managed with sertraline and prazosin. The patient was only partially compliant with medications.

The patient was inpatient for 16 days. He was placed on mirtazapine, sertraline, and prazosin. Psychotherapy was started in the inpatient unit, and the patient was referred for further therapy on discharge.

While the short inpatient stays mostly addressed immediate stabilization, both patients verbalized relief from psychoeducation about their illness.

## 3. Discussion

Recognizing and treating PCBD appropriately cannot be overemphasized. In both of our patients, alternative and comorbid diagnoses of MDD and PTSD were recognized while PCBD was missed. While PCBD can be comorbid with other psychiatric disorders, it is known to be a unique response to loss with its accompanying suffering [15, 16]. PCBD and MDD may share symptoms like sadness, crying, guilt, and suicidal thinking, but with PCBD, the focus is on the loss [1]. In terms of PTSD, loss related to traumatic death can result in both PTSD and PCBD. Such situations may involve both intrusive thoughts and avoidance. Intrusions in PTSD are usually about the event leading to the death, while the intrusive thoughts in PCBD are focused on the relationship with the deceased and distress over the loss [1]. Notably, PCBD has a traumatic bereavement specifier which applies to preoccupation with the nature of death [1]. Avoidance of reminders of distressing events can occur in both PTSD and PCBD. In PTSD, there is consistent avoidance of the internal and external reminders of the traumatic experience, whereas in PCBD, there is a preoccupation with the loss and a yearning for the deceased [1].

PCBD is also significantly associated with substance use disorders (SUD) [17]. Both of our patients presented with SUD. Masferrer et al. [18] identified 4 factors including discomfort, nonacceptance, loneliness-isolation, and the presence of the deceased as associated with substance use in bereaved drug-dependent individuals. Of note, nonacceptance was not found in another study with bereaved individuals without SUD [18]. Therefore, substance use might be a measure to avoid the reality of grief. Hamden et al. [19] showed that bereaved youth are at increased risk of substance use compared to nonbereaved controls mainly due to their poor functional status postbereavement. For patients who experience significant loss early in life like patient XY, early disruption in care may alter their response to later life stressors therefore predisposing them to substance use [17]. Individuals with cooccurring MDD, PTSD, and anxiety disorders with SUD have worse treatment outcomes for SUD and are more likely to relapse [20]. Similarly, screening for and treating grief-related problems among SUD patients has shown promising results [21].

The most important risk factors for PCBD are the nature of death [22, 23] and the nature of the relationship [16]. First-degree relationships like loss of a child or spouse carry an increased risk [22, 23]. Likewise, deaths due to cardiac disease or stroke, suicide, trauma, cancer, and spending extensive time with the deceased in the last week of life have a higher risk [22, 23]. Both of our patients lost first-degree relatives to cancer, and they were with their loved ones through the periods of suffering and death. Additionally, both patients reported having very close relationships with their loved ones before they died. The premorbid psychiatric condition of patients is also very important, especially the presence of affective disorders [6]. Patient AB had a prior diagnosis of MDD as a teenager. Availability of social support has been shown to be a significant periloss factor [6]; however, in our patients, social support did not seem to make a difference. AB had significant social support while patient XY did not.

Neurobiologically, findings have indicated possible deficits in the stress response and neural reward/attachment systems [24]. O'Connor et al. [24] showed that yearning is associated with the activation of the reward pathways especially the nucleus accumbens in patients with PCBD; this craving-like behavior that is similar to what occurs in an addiction prevents a normal loss response. It has been suggested that perhaps the avoidance behavior in PCBD may be a way of managing the reward response produced by perpetual yearning [25]. In addition, PCBD patients compared to bereaved patients without PCBD have shown reduced activity in the orbitofrontal cortices and delayed recruitment of the dorsal anterior cingulate when exposed to grief-related stimuli [25]. This reduced activity may be responsible for possible deficits in emotion regulation in patients with PCBD [25]. LeBlanc et al. [26] suggested possible emotional inflexibility secondary to blunted parasympathetic nervous system reactivity in PCBD patients. Finally, individuals with higher grief severity have been shown to have higher levels of proinflammatory cytokines (interferon-*γ*, interleukin-6, and tumor necrosis factor-*α*) suggesting a role for immune and inflammatory systems [27].

In our patients, PCBD was diagnosed using the DSM-5 criteria as shown in Table 2; however, there are other measures used to screen patients for complicated grief. They include the Inventory of Complicated Grief, the Brief Screen for Complicated Grief, and the Prolonged Grief Disorder scales [6, 28]. These scales are self-administered, and a calculation of the scores post completion indicates if PCBD is present or not. As an alternative, Bui et al. [28] developed a clinician-administered Structured Clinical Interview to assess the presence and severity of PCBD.

Treatment-wise, for pharmacotherapy, there are no FDA-approved medications, and generally in literature, there is no consensus on medications that are helpful in the treatment of PCBD. Modest results have been demonstrated in the treatment with escitalopram [6], bupropion [29], paroxetine [6, 29], and nortriptyline [29]; however, all of these studies emphasized the need for more studies and the possibility that the effects of these medications were due to the treatment of comorbid depression [29]. Psychotherapy is the mainstay for the treatment of PCBD. Complicated Grief Therapy, a targeted therapy focused on resolving complications and facilitating adaptation to loss, has been shown to be effective [6].

CGT has elements of cognitive behavioral therapy and interpersonal therapy [6]. It also includes psychoeducation and motivational interviewing [6]. It focuses on coming to terms with loss and restoration of function [30]. In a randomized clinical trial, CGT was more effective than placebo in improving the outcome of PCBD and reducing suicidal ideation [31]. In the same study, citalopram was not effective against placebo, but the addition of citalopram to CGT reduced the depressive symptoms [31]. In another study, CGT had a higher response rate and faster time to response when compared to interpersonal psychotherapy for the treatment of PCBD [32].

## 4. Conclusion

Screening for PCBD, using the screening tools mentioned earlier, should be included in the care of bereaved patients especially those with significant risk factors, treatment-resistant psychiatric disorders, and those who present for emergency treatment due to comorbidities. In addition, when recognized, PCBD should be promptly treated to reduce morbidity in these patients.

## Figures and Tables

**Table 1:** Risk factors for Persistent Complex Bereavement Disorder [6, 13]. Superscripts show risk factors that were present in patients AB and XY.

Preloss factors	Loss-related factors	Periloss factors
(i) Female sex^AB^ (ii) Preexisting trauma (particularly childhood trauma) (iii) Prior loss (iv) Insecure attachment^XY^ (v) Poorly functioning marriage (vi) Separation anxiety in childhood (vii) Preexisting mood and anxiety disorders^AB^ (viii) Nature of the relationship—first degree relationship^AB,XY^	(i) Relationship and caretaking roles: spouses, mothers of dependent children, caretakers for chronically ill^AB^ (ii) Nature of the death itself: violent, sudden, prolonged, due to suicide, in-hospital death of the loved one^AB,XY^ (iii) Lack of preparation for the death^AB,XY^	(i) Social circumstances (ii) Resources available following death^XY^ (iii) Poor understanding of the circumstances of the death event, i.e., lack of information about the death (iv) Interference with natural healing process: inability to follow usual cultural practices of death and mourning, alcohol or substance use^AB,XY^ (v) Poor social support^XY^

**Table 2:** Diagnostic criteria for Persistent Complex Bereavement Disorder in the DSM-5. Patients with PCBD should endorse at least one separation distress symptom and six additional symptoms. Superscripts show criteria that were present in patients AB and XY.

Separation distress	Reactive distress to death	Social/identity disruption
(i) Persistent yearning/longing for the deceased^AB,XY^ (ii) Intense sorrow and emotional pain in response to the death^AB,XY^ (iii) Preoccupation with the deceased^AB,XY^ (iv) Preoccupation with the circumstances of the death^AB,XY^	(i) Marked difficulty accepting the death^AB,XY^ (ii) Experiencing disbelief or emotional numbness over the loss (iii) Difficulty with positive reminiscing about the deceased^AB,XY^ (iv) Bitterness or anger related to the loss^AB,XY^ (v) Maladaptive appraisals about oneself in relation to the deceased or the death (e.g., self-blame)^AB,XY^ (vi) Excessive avoidance of reminders of the loss^AB^	(i) A desire to die in order to be with the deceased (ii) Difficulty trusting other individuals since the death^AB,XY^ (iii) Feeling alone or detached from other individuals since the death^AB,XY^ (iv) Feeling that life is meaningless or empty without the deceased or the belief that one cannot function without the deceased^AB,XY^ (v) Confusion about one's role in life or a diminished sense of one's identity (e.g., feeling that a part of oneself died with the deceased)^AB,XY^ (vi) Difficulty or reluctance to pursue interests since the loss or to plan for the future^AB,XY^
